# Seizures and premature death in mice with targeted Kv1.1 deficiency in corticolimbic circuits

**DOI:** 10.1093/braincomms/fcae444

**Published:** 2025-01-16

**Authors:** Kelsey Paulhus, Edward Glasscock

**Affiliations:** Department of Biological Sciences, Southern Methodist University, Dallas, TX 75275, USA; Department of Biological Sciences, Southern Methodist University, Dallas, TX 75275, USA

**Keywords:** potassium channel, apnoea, heart rate variability, epilepsy comorbidity

## Abstract

Sudden unexpected death in epilepsy (SUDEP) is the leading cause of epilepsy-related death, likely stemming from seizure activity disrupting vital brain centres controlling heart and breathing function. However, understanding of SUDEP's anatomical basis and mechanisms remains limited, hampering risk evaluation and prevention strategies. Prior studies using a neuron-specific *Kcna1* conditional knockout mouse model of SUDEP identified the primary importance of brain-driven mechanisms contributing to sudden death and cardiorespiratory dysregulation; yet, the underlying neurocircuits have not been identified. Using the *Emx1*-Cre driver, we generated a new conditional knockout mouse model lacking *Kcna1* in excitatory neurons of the cortex, hippocampus, amygdala and select vagal afferents. To test whether the absence of Kv1.1 in forebrain corticolimbic circuits is sufficient to induce spontaneous seizures, premature mortality and cardiorespiratory dysfunction, we performed survival studies and EEG, ECG, and plethysmography (EEG-ECG-Pleth) recordings. We demonstrate premature death and epilepsy in corticolimbic conditional knockout mice. During monitoring, we fortuitously captured one SUDEP event, which showed a generalized tonic-clonic seizure that initiated respiratory dysfunction culminating in cardiorespiratory failure. In addition, we observed that cardiorespiratory abnormalities are common during non-fatal seizures in conditional knockout mice, but mostly absent during interictal periods, implying ictal, not interictal, cardiorespiratory impairment as a more reliable indicator of SUDEP risk. These results point to corticolimbic excitatory neurons as critical neural substrates in SUDEP and affirm seizure-related respiratory and cardiac failure as a likely cause of death.

## Teaser

Paulhus and Glasscock use conditional knockout mice to show corticolimbic Kv1.1 deficiency leads to premature death and seizures with cardiorespiratory dysfunction. They describe a sudden unexpected death in epilepsy event with seizure-initiated respiratory dysfunction culminating in cardiorespiratory failure, highlighting the potential importance of corticolimbic neurocircuits as critical sudden death in epilepsy substrates.

## Graphical Abstract

**Graphical Abstract:**
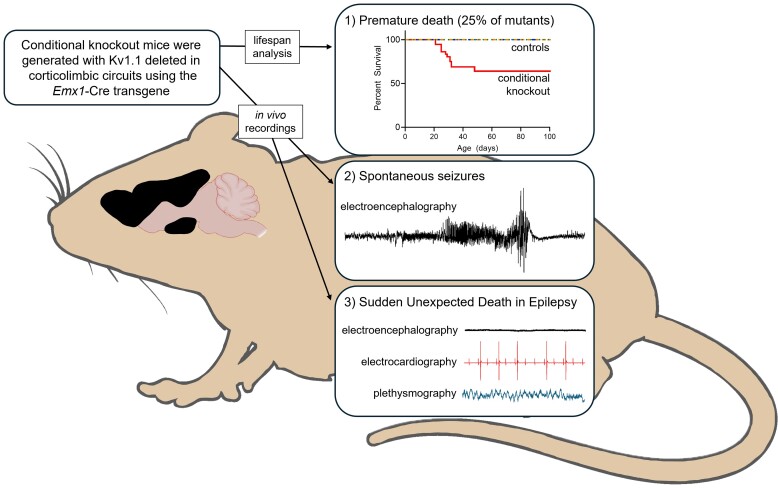

## Introduction

Sudden unexpected death in epilepsy (SUDEP) is the leading cause of epilepsy-related mortality.^1,2^ Besides having epilepsy, affected individuals are generally healthy and show no accidental, pathological, or toxicological explanation for their death.^3,4^ The current view of SUDEP is that seizures somehow trigger deleterious cardiac and respiratory dysfunction that culminates in death.^5^ However, the exact details of how neural, cardiac, and respiratory factors contribute to this catastrophic outcome remain unresolved.

Contemporary clinical understanding of SUDEP is based largely on the MORTEMUS study (MORTality in Epilepsy Monitoring Units Study), a description of 9 rare cases that were captured in epilepsy monitoring units by video-EEG and ECG and retrospectively analysed for terminal seizure and cardiorespiratory patterns.^6^ These cases exhibited a pattern of generalized tonic-clonic seizures followed by post-ictal increases in respiratory and heart rates (HRs), progressing to a lethal combination of central apnoea, bradycardia and asystole, coinciding with post-ictal generalized EEG suppression (PGES).^6^ Terminal apnoea always preceded cardiac arrest, implying the potential primacy of respiratory mechanisms.^6^ However, whether these few observed cases are representative of all SUDEP remains to be seen.

Much of what is known about SUDEP pathomechanisms and the underlying anatomy comes from work in animals.^7^ One of the most widely used SUDEP models is the *Kcna1* global knockout (KO) mouse because it exhibits essential features observed in humans.^8^ These characteristics include frequent generalized tonic-clonic seizures, seizure-related sudden death, and ictal cardiorespiratory dysfunction with aberrant breathing occurring before cardiac abnormalities. The *Kcna1* gene encodes pore-forming Kv1.1 voltage-gated potassium channel α-subunits and has direct genetic relevance for human epilepsy and SUDEP. At least 21 different human *KCNA1* mutations have been identified that cause epilepsy, including four associated with epileptic encephalopathy, which carries high risk of SUDEP.^9^ Building upon findings in *Kcna1* global KO mice, previously we generated neuron-specific *Kcna1* conditional knockout mice to test whether seizure-evoked cardiorespiratory dysfunction and SUDEP require the absence of Kv1.1 in both brain and heart or whether ablation in neurons is sufficient.^10^ We found that deletion of Kv1.1 in neurons alone was sufficient to cause epilepsy, premature death, and cardiorespiratory dysregulation. However, the type and location of these neurons remain unknown.

To begin mapping neuronal subpopulations contributing to SUDEP, here we generated conditional knockout mice lacking Kv1.1 in forebrain circuits, specifically excitatory neurons of the neocortex, hippocampus, and amygdala where Kv1.1 is normally highly abundant.^11^ We then recorded EEG, ECG, and plethysmography in conditional knockout mice to identify seizures and cardiorespiratory abnormalities. We find that corticolimbic deletion of *Kcna1* leads to spontaneous seizures, premature mortality, and ictal cardiorespiratory dysfunction. We also report a captured SUDEP event, which revealed terminal cardiorespiratory patterns that resemble previous descriptions in humans. Our results point to excitatory neurons within corticolimbic circuits as critical drivers of epilepsy and subsequent SUDEP due to cardiorespiratory failure. These findings provide the first identification of the brain regions underlying seizures and SUDEP in the popular *Kcna1* KO mouse model, as well as one of the only observations of a SUDEP event with cardiorespiratory data revealing the terminal sequence of events.

## Materials and methods

### Animals and genotyping

Corticolimbic *Kcna1* conditional knockout mice (i.e. *Emx1*-Cre^+/−^; *Kcna1^del/^*^−^) were generated by crossing heterozygous *Kcna1* floxed (fl) mice (*Kcna1^fl/^*^+^) with heterozygous *Kcna1* global knockout mice (*Kcna1*^+/−^) carrying one copy of the *Emx1*-Cre transgene (i.e. *Emx1*-Cre^+/−^, *Kcna1*^+/−^). *Emx1*-Cre^+/−^, *Kcna1*^+/−^ mice were generated by crossing hemizygous transgenic *Emx1*-Cre^+/−^ mice with heterozygous *Kcna1*^+/−^ mice. The *Emx1*-Cre^+/−^ transgene causes Cre-mediated recombination of the *Kcna1^fl^* allele to yield the conditionally deleted allele (i.e. *Kcna1^del^*). This breeding paradigm, which has been utilized in previous studies,^10^ was selected because it prevents unwanted Cre-mediated germline recombination and promotes increased Cre-mediated recombination efficiency. The above breeding scheme also yields the following mice which were designated as control genotypes for experiments: *Kcna1*^+/+^, *Emx1*-Cre^−/−^ [wild type (WT)], *Kcna1*^+/+^, *Emx1*-Cre^+/−^ (Cre), and *Kcna1*^fl/−^, *Emx1*-Cre^+/+^ (fl/-). *Emx1*-Cre mice were purchased from Jackson Labs (Bangor, ME) under the catalog name B6.129S2-*Emx1^tm1(cre)Krj^*/J (JAX 005628). *Kcna1*^+/−^ mice, which are maintained on a Tac:N:NIHS-BC genetic background, carry a null allele of the *Kcna1* gene due to targeted deletion of the entire open reading frame.^8^  *Kcna1*^fl/+^ mice, which are maintained on a C57BL/6J genetic background, were generated as described previously.^10^ Mice were housed at 22°C, fed *ad libitum* and subjected to a 12-h light/dark cycle. For lifespan analysis, both experimental and control mice were monitored for mortality until the age of 100 days. All experiments were performed in accordance with National Institutes of Health (NIH) guidelines with approval from the Institutional Animal Care and Use Committee of Southern Methodist University.

To identify experimental and control mice, genomic DNA was isolated by enzymatic digestion of tail clips using Direct-PCR Lysis Reagent (Viagen Biotech, Los Angeles, CA, USA). Genotypes were determined by performing PCR amplification of genomic DNA using allele-specific primers. The primer sequences used for amplifying the various *Kcna1* alleles have been described previously.^10^ For detection of the *Emx1*-Cre transgene, the following primer sequences were used to yield a product of 315 bp for the WT allele, and 195 bp for the Cre allele: a WT-specific primer (5′-CAAAGACAGAGACATGGAGAGC-3′), a *Emx1*-Cre specific primer (5′-TCGATAAGCCAGGGGTTC3′) and a common primer (5′-CAACGGGGAGGACATTGA-3′).

### Western blotting

To isolate and quantify protein levels of Kv1.1, age- and sex-matched mice (1–2 months old) were euthanized by isoflurane overdose, and their brains were quickly removed and dissected on ice to separate the cortex, hippocampus and brainstem. Tissues were then processed, and immunoblotting performed as described previously but with some minor modifications listed below.^10^ Protein concentrations of the brain region homogenates were determined using bicinchoninic acid assay (Thermo Scientific; Waltham, MA, USA). The primary antibody solution was prepared in Tris-buffered saline with Tween (0.1% v/v) with bovine serum albumin (5% w/v). The primary antibodies used were mouse monoclonal anti-Kv1.1 (1:500; K20/78; Antibodies Incorporated; Davis, CA, USA) and mouse monoclonal anti-β-actin (1:500, sc-47778, Santa Cruz Biotechnology; Dallas, TX, USA). Immunoreactive bands were visualized using an enhanced chemiluminescence detection kit (ImmunoCruz, Santa Cruz Biotechnology; Dallas, TX, USA) and developed on a UVP ChemStudio imaging system (Analytik Jena; Upland, CA, USA). Band intensity was quantified using densitometry analysis on VisionWorks software (Analytik Jena; Upland, CA, USA), normalized to β-actin levels, and reported as relative intensity.

### Video-EEG-ECG-plethysmography recordings

To record *in vivo* brain and heart activity, mice (4–6 weeks old) of both sexes were anaesthetized using an anaesthetic cocktail and surgically implanted with bilateral silver wire EEG and ECG electrodes (0.005-inch diameter) attached to a microminiature connector (Omnetics Connector Corporation, Minneapolis, MN, USA) for recording in a tethered configuration, as described previously.^10,12^ Briefly, EEG wires were inserted into the subdural space through cranial burr holes overlying the parietotemporal cortex for the recording electrodes and above the frontal cortex for the ground and reference electrodes. The two ECG wires were tunneled subcutaneously on both sides of the thorax and sutured in place to record cardiac activity. Mice were allowed to recover for 48 h before recording simultaneous EEG-ECG for 24 h continuously.

For recording respiratory waveforms in tandem with EEG-ECG, mice were placed in an unrestrained whole-body plethysmography (pleth) chamber (Data Sciences International, St. Paul, MN, USA) with a lid that was modified to accommodate wires for recording EEG-ECG in a tethered configuration, as described previously.^13^ Mice were provided a 45-min acclimatization period in the chamber before video and EEG-ECG-pleth were simultaneously recorded in 5–6 h sessions during the light phase of the day (i.e. between 6:00 a.m. and 6:00 p.m.) using Ponemah data acquisition and analysis software (Data Sciences International, St. Paul, MN, USA). Biosignals were filtered and sampled as reported previously.^10,13^

### Analysis of video-EEG, ECG and pleth recordings

Seizures and cardiorespiratory abnormalities were identified by visual inspection of the biosignals as described previously.^10,13^ Behavioural and cardiorespiratory phenotypes during seizures were manually classified from video footage and quantified as the percentage of seizures manifesting the specific behavior. Quantification of cardiac and respiratory features was performed using Ponemah software (Data Sciences International, St. Paul, MN, USA) as described previously.^10,13^ Briefly, for estimating HR and HR variability, six separate RR interval series were derived by sampling 2-min ECG segments every 4 h to provide three light phase (6:00 a.m. to 6:00 p.m.) and three dark phase (6:00 p.m. to 6:00 a.m.) measurements. The HR and HR variability values for each animal were then averaged from the six total segments. RR intervals were only sampled during times when the mouse was stationary and when the ECG showed no abnormalities such as skipped or ectopic beats occurred. Skipped beats (e.g. cardiac conduction blocks or sinus pauses) were identified from the ECG waveforms and defined as a prolongation of the RR interval equaling ≥ 1.5 times the previous RR interval.

Data for respiratory rate and variability measurements were collected during periods when animals were stationary, as verified by video monitoring. Respiratory variability was calculated as the coefficient of variance using the formula: coefficient of variance = σ/µ, where σ is the standard deviation of breath intervals and µ is mean of breath intervals. Apnoeas were identified as cessations of plethysmographic signals for ≥ 2 respiratory cycles, or 0.8-s, as done previously.^13^ Abnormal patterns of respiration, characterized by complete irregularity in breath frequency resembling ataxic breathing, were classified as ataxic-like breathing patterns to account for the possibility of movement artefacts influencing the waveforms. For cardiac and respiratory measures related to rate, tachy- and brady-events were defined as rate changes of at least +20% or −20% respectively, relative to the 20-s pre-ictal period immediately before seizure onset as done previously.^13^ Pre- and post-ictal phenotypes were counted if they occurred in the 20-s before seizure onset or after its termination, respectively.

### Statistical analysis and blinding

Data are presented as mean ± SEM. Prism 10 for Windows (GraphPad Software Inc, La Jolla, CA, USA) was used for statistical analysis. Survival curves were evaluated using the Kaplan-Meier log rank (Mantel-Cox) test. For comparisons involving two or more groups, one-way analysis of variance was used followed by Tukey *post hoc* tests. Differences between groups were deemed statistically significant if *P* < 0.05. Analysis of biosignal recordings was performed in a blinded fashion by naming files in a manner that obscured genotype.

## Results

### Molecular characterization of corticolimbic *Kcna1* conditional knockout mice

**Figure 1:**
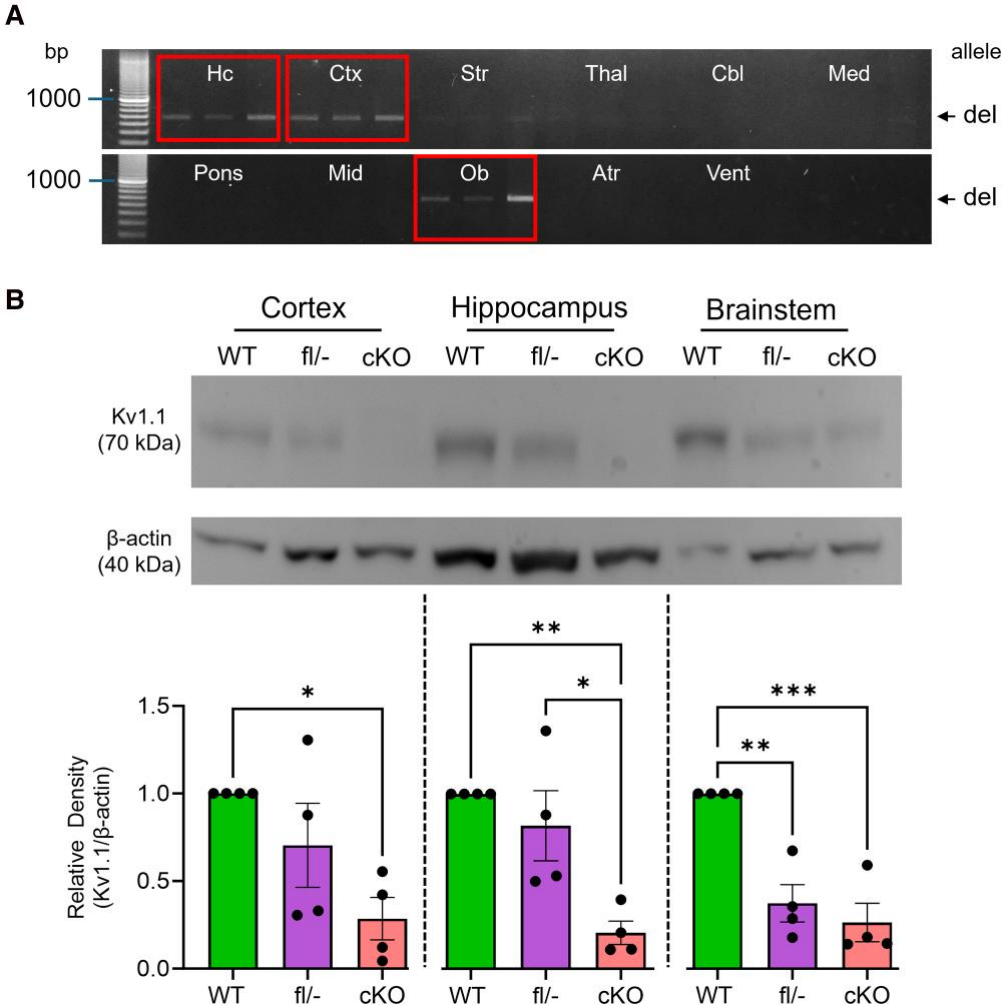
**Molecular characterization of corticolimbic *Kcna1* conditional knockout mice.** (**A**) PCR detection of the *Kcna1* deletion (del; 679 bp) allele from genomic DNA isolated from hippocampus (Hc), cortex (Ctx), striatum (Str), thalamus (Thal), cerebellum (Cbl), medulla (Med), pons, midbrain (Mid), olfactory bulb (Ob), left atrium (Atr) and left ventricle (Vent) for conditional knockout (cKO) mice. The three lanes for each region represent a different corticolimbic conditional knockout mouse (*n* = 3 cKO mice per region: 2 males, 1 female). Boxes indicate regions where the deletion band is observed at a high level. (**B**) Representative western blots for Kv1.1 and β-actin loading control from the cortex, hippocampus and brainstem from WT (*n* = 4: 1 male, 3 females), *Kcna1*^flox/−^ (fl/−; *n* = 4: 3 males, 1 female) and corticolimbic *Kcna1* conditional knockout mice (*n* = 4: 3 males, 1 female, 1–2 months old) with corresponding quantification of relative density for Kv1.1 protein levels normalized to β-actin (*n* = 4/genotype). Each data point represents a single animal. *P < 0.05, **P < 0.01, ***P < 0.001; one-way ANOVA. The full uncropped gel in **A** and blot in **B** are included as Supplementary Figs. 1 and 2, respectively.

We generated corticolimbic *Kcna1* conditional knockout mice (i.e. *Emx1*-Cre^+/−^; *Kcna1^del/^*^−^) using the breeding scheme outlined in the Materials and Methods. The *Emx1*-Cre driver is a commonly used transgene that selectively targets most excitatory neurons in the neocortex and hippocampus; olfactory bulb; regions of the amygdala, including the basolateral, basomedial, lateral, posterolateral cortical and posteromedial amygdaloid nuclei; and select vagal afferents.^14-17^ To confirm the tissue-specific deletion of *Kcna1*, we performed PCR and immunoblots of brains from conditional knockout and control mice. PCR amplification of regionally dissected brain tissue revealed the presence of the *Kcna1^del^* allele in the neocortex, hippocampus and olfactory bulb, but not in the brainstem (e.g. medulla, pons and midbrain), cerebellum or heart (Fig. 1A). We next performed Western blots to measure Kv1.1 protein levels in the brains of conditional knockout mice. Immunoblots showed significantly reduced levels of Kv1.1 in the neocortex and hippocampus, as compared with WT, consistent with the reported expression pattern of the *Emx1*-Cre transgene (Fig. 1B). The low levels of residual Kv1.1 likely stem from its presence in other cell types that are not targeted by *Emx1*-Cre, such as inhibitory neurons. In the brainstem, which is unaffected by *Emx1*-Cre targeting, conditional knockout mice exhibited Kv1.1 levels comparable to those observed in *Kcna1^fl^*^/−^ mice. This similarity was expected, as both types of mice carry one copy of the global KO null allele which should reduce Kv1.1 abundance by about half.

### Corticolimbic conditional knockout mice die prematurely

**Figure 2:**
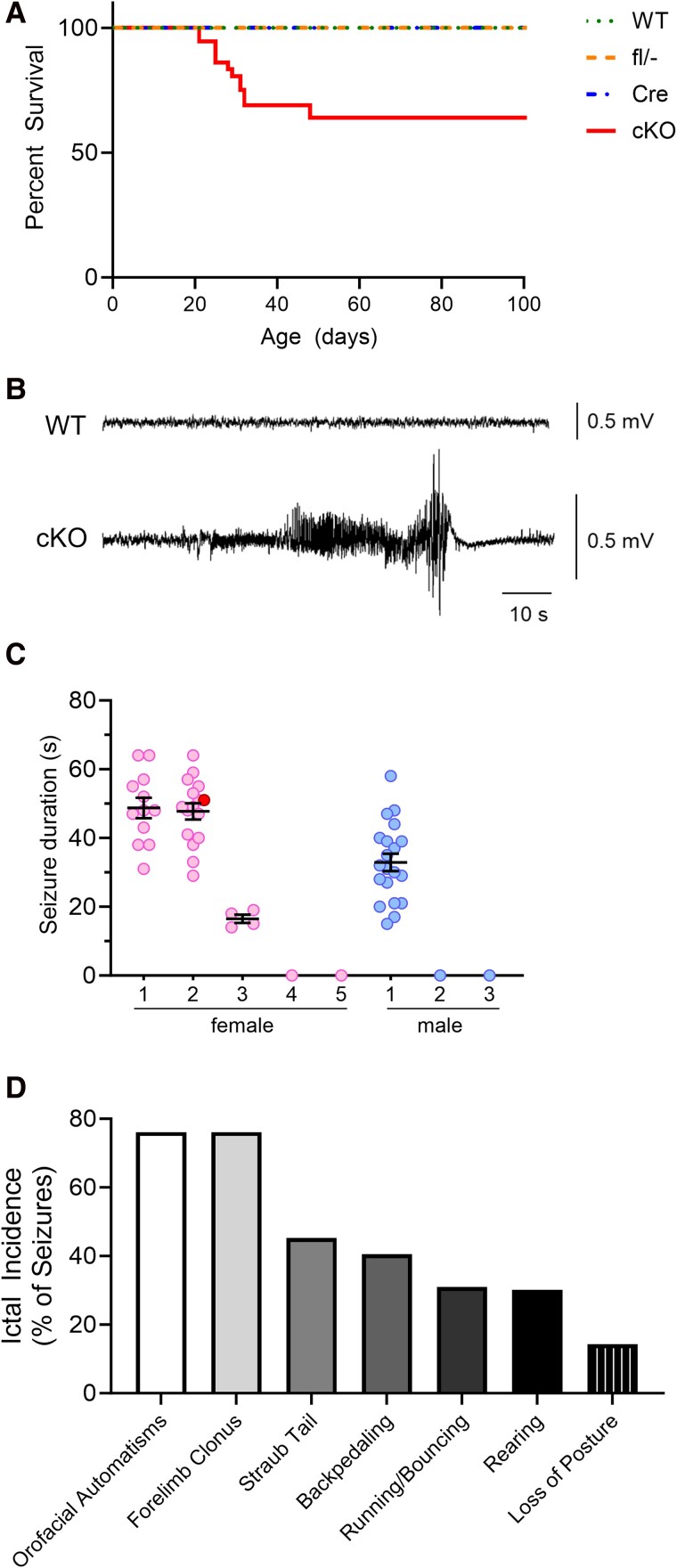
**Corticolimbic *Kcna1* conditional knockout (cKO) mice exhibit premature death and spontaneous seizures.** (**A**) Kaplan–Meier survival curves showing lifespan for WT (*n* = 20), hemizygous *Emx1*-Cre (Cre; *n* = 41), compound heterozygous *Kcna1*^fl/−^ (fl/-; *n* = 72), and corticolimbic cKO (*n* = 36) mice. In cKO, survival was significantly decreased compared with Cre, fl/− and WT controls (*P* < 0.0001, Log-rank test). (**B**) Representative EEG traces from a WT and cKO mouse showing normal and seizure activity, respectively. (**C**) Plot of individual seizure durations for every seizure recorded in each cKO animal (5 females and 3 males) in the study. Female mouse number two died from SUDEP during the recording, and the terminal seizure is indicated by the darker shaded circle. (**D**) Frequency of various seizure-associated behaviours in cKO mice, quantified as the percentage of total seizures in which they occurred (*n* = 52 seizures in 4 cKO mice: 1 male, 3 females).

To assess whether Kv1.1 deficiency in corticolimbic circuits contributes to premature mortality, we measured postnatal survival during the first 100 days of life. Corticolimbic conditional knockout mice showed significant premature mortality with 25% of animals dying between 3 to 7 weeks of age (Fig. 2A; *P* < 0.0001, Log-Rank test). Nearly all early deaths occurred between 3 and 4 weeks old, with the youngest age at death being 21 days old. Importantly, corticolimbic conditional knockout mice that experienced premature mortality were found in a state of tonic hindlimb extension, consistent with death immediately following a seizure. In contrast, control mice all lived to 100-day old, showing no evidence of early lethality (Fig. 2A). Thus, deletion of *Kcna1* in excitatory corticolimbic neurons contributes to premature sudden death.

### Corticolimbic conditional knockout mice exhibit spontaneous seizures

To test for the occurrence of spontaneous seizures, we performed continuous video-EEG-ECG recordings for 24 h, followed by an additional 6 h of video-EEG-ECG-pleth recordings. EEG analysis revealed the presence of spontaneous seizures in four of eight (50%) conditional knockout mice during the recording period but none in control mice (Fig. 2B and C). The EEG patterns typically consisted of runs of polyspike activity with progressively increasing amplitude followed by post-ictal flattening (Fig. 2B). Immediately following seizure termination, about 80% of seizures exhibited PGES characterized by prolonged flattening of EEG activity lasting for several seconds. For the conditional knockout mice that exhibited seizures, the average seizure frequency was 0.5 ± 0.1/h with an average seizure duration of 40 ± 14 s (Fig. 2C). Of the 52 individual seizures recorded in conditional knockout mice, 5.8% (3/52) lasted > 60 s.

Using the corresponding video recordings, we also analysed the behaviours associated with the EEG seizures (Fig. 2D). Most seizures (75%) exhibited milder behavioural manifestations, such as orofacial automatisms and forelimb clonus. However, we also observed more severe convulsive behaviours, such as running and bouncing, rearing and loss of posture, but those behaviours occurred less frequently in about 15–30% of seizures. Thus, Kv1.1 deficiency in corticolimbic circuits contributes to spontaneous generalized tonic-clonic seizures in mice.

### Corticolimbic conditional knockout mice exhibit seizure-related cardiac dysfunction

**Figure 3:**
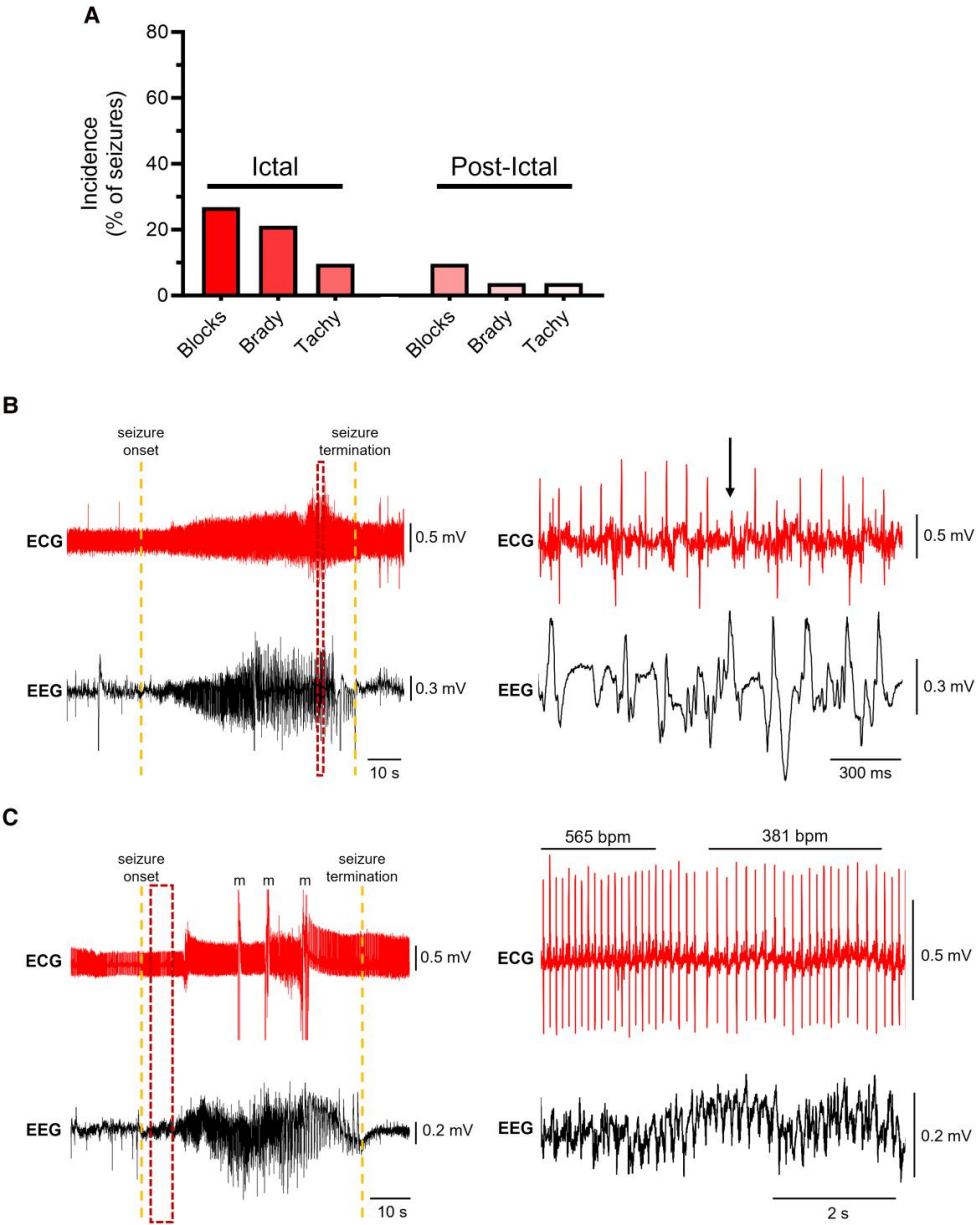
**Corticolimbic *Kcna1* conditional knockout mice exhibit ictal cardiac dysfunction.** (**A**) Quantification of the incidence of various cardiac abnormalities during seizures (ictal) and post-ictal periods. The bars represent the percentage of total seizures in which they occurred (*n* = 52 seizures in 4 conditional knockout mice: 1 male, 3 females; 2–18 seizures per animal). Blocks = cardiac conduction blocks or sinus pauses; Brady = bradycardia; Tachy = tachycardia. (**B**) Representative EEG-ECG recording of a seizure with an ictal cardiac conduction block or sinus pause. The left panel displays the full-length EEG seizure and the corresponding ECG activity, with seizure onset and termination marked by the dotted lines. The right panel provides an expanded view of the area in the dashed box from the left panel, highlighting the conduction block or sinus pause, which is indicated by an arrow. (**C**) Representative EEG-ECG recording of a seizure with ictal bradycardia. The left panel displays the full-length EEG seizure and the corresponding ECG activity, with seizure onset and termination marked by the dotted lines. Movement artefacts (m) are indicated in the ECG trace. The right panel provides an expanded view of the area in the dashed box from the left panel, highlighting the bradycardia event, which is indicated by bars above the corresponding ECG segments with the rates labeled in beats per minute (bpm).

To determine whether conditional knockout mice exhibit ictal or post-ictal cardiac dysfunction, ECG recordings were analysed during and immediately after seizures. We found that seizures evoked a variety of cardiac abnormalities ranging from cardiac conduction blocks/sinus pauses to rhythm disturbances (Fig. 3A). Cardiac conduction blocks or sinus pauses (i.e. skipped heart beats) were the most common cardiac abnormality observed during seizures, occurring in 27% of seizures (Fig. 3B). The second most common cardiac phenotype was bradycardia, which was present in 23% of seizures (Fig. 3C). We also observed ictal tachycardia, but it was rarer occurring in <10% of seizures (Fig. 3A). During post-ictal periods, cardiac dysfunction was relatively infrequent with cardiac conduction blocks or sinus pauses, bradycardia and tachycardia occurring after 4–10% of seizures (Fig. 3A). Thus, *Kcna1* deficiency in corticolimbic circuits is associated with ictal and post-ictal cardiac dysfunction.

### Corticolimbic conditional knockout mice exhibit seizure-related respiratory dysfunction

**Figure 4:**
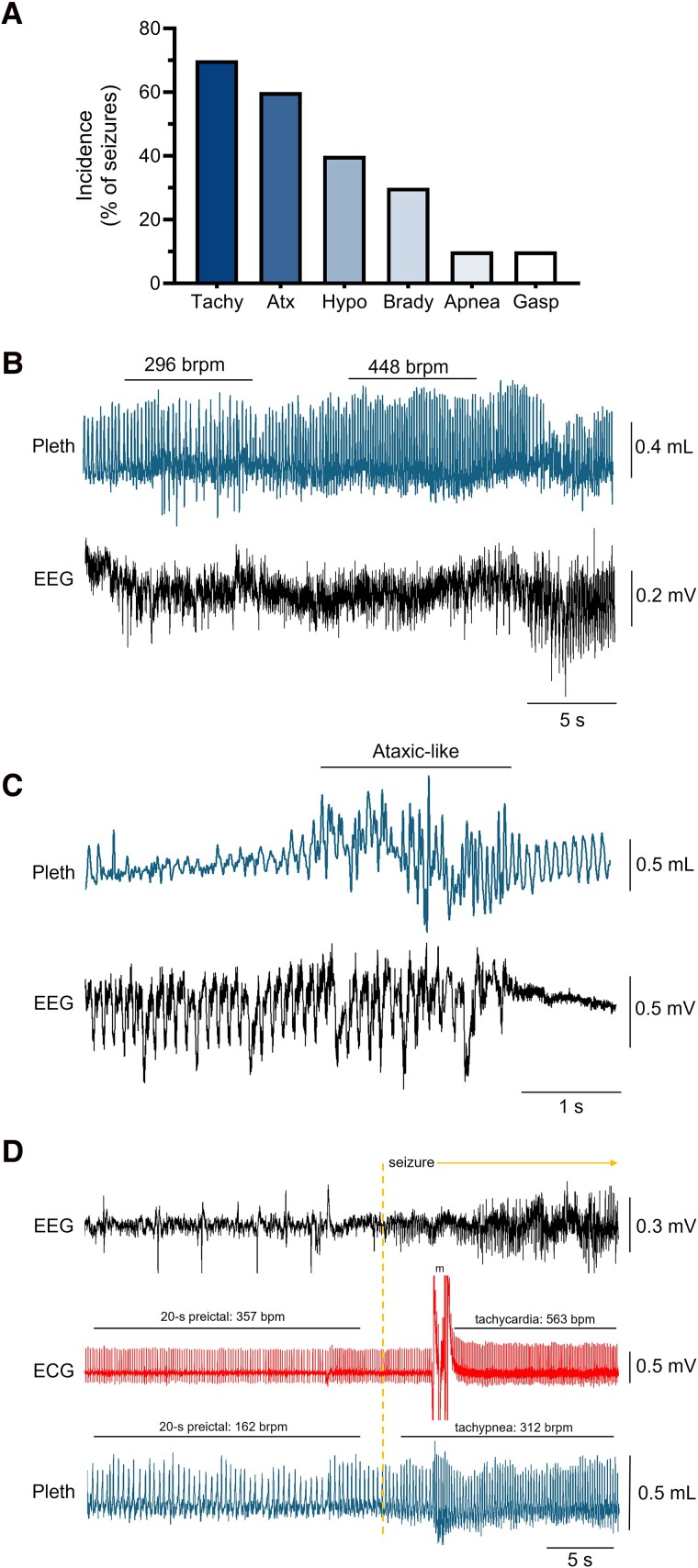
**Corticolimbic *Kcna1* conditional knockout mice exhibit ictal respiratory dysfunction.** (**A**) Quantification of the incidence of various respiratory abnormalities during seizures. The bars represent the percentage of total seizures in which they occurred (*n* = 10 seizures in 3 conditional knockout mice: 1 male, 2 females; 2–6 seizures per animal). Tachy = tachypnea; Atx = ataxic-like breathing patterns; Hypo = hypopnea; Brady = bradypnea; Gasp = gasping. (**B**) Representative plethysmography (pleth) trace of ictal tachypnea with corresponding EEG seizure activity. The breathing rates before (left) and during (right) the tachypnea event are indicated by bars above the corresponding pleth segments with the rates labeled in breaths per minute (brpm). (**C**) Representative pleth trace showing ictal ataxic-like breathing patterns (indicated by the labeled bar) with corresponding EEG seizure activity. (**D**) Representative EEG-ECG-pleth recording of a seizure that initiates ictal tachypnea before ictal tachycardia. The dotted line marks onset of the seizure. Heart rate (bpm, beats per minute) and breathing rate (brpm, breaths per minute) are labeled above the corresponding ECG and pleth segments, respectively. As outlined in the Materials and methods, tachycardia and tachypnea were defined as rate increases of at least 20% relative to the 20-s pre-ictal period immediately prior to seizure onset. Movement artefacts (m) are indicated in the ECG trace.

To examine whether corticolimbic Kv1.1 deficiency is associated with respiratory dysregulation during seizures, we analysed the whole-body plethysmography data from the 6-h video-EEG-ECG-pleth recordings. During seizures, respiratory dysfunction occurred more often than cardiac dysfunction. The most common ictal respiratory phenotypes were tachypnea and ataxic-like breathing patterns, which occurred in 70% and 60% of seizures, respectively (Fig. 4A–C). Of note, when both ictal respiratory and cardiac dysfunction were present, respiratory dysfunction occurred first in 80% (8/10) of seizures (Fig. 4D) and appeared concurrently with cardiac dysfunction in 20% (2/10) of seizures. Interestingly, while global KO and neuron-specific conditional knockout mice reliably display hyperventilation/tachycardia at seizure onset,^10,13^ the corticolimbic conditional knockout mice did not consistently exhibit this phenotype. These findings suggest that the absence of Kv1.1 in corticolimbic circuits contributes to seizures that impair breathing, which may lead to cardiac dysfunction and an increased risk of SUDEP.

### Corticolimbic conditional knockout mice exhibit SUDEP due to seizure-evoked cardiorespiratory dysfunction

During EEG-ECG-pleth monitoring, we fortuitously captured a SUDEP event in a 30-day-old female conditional knockout mouse (labeled conditional knockout #2 in Fig. 2C), providing critical insight into the circumstances leading up to and during the terminal seizure. In the 24 h preceding the terminal seizure, the mouse exhibited 15 seizures, the second most seizures of any conditional knockout mouse. Furthermore, the seizures had averaged 47 ± 9 s in duration, the second longest durations of any conditional knockout (Fig. 2C), suggesting the animal’s seizure disorder was particularly severe.

**Figure 5:**
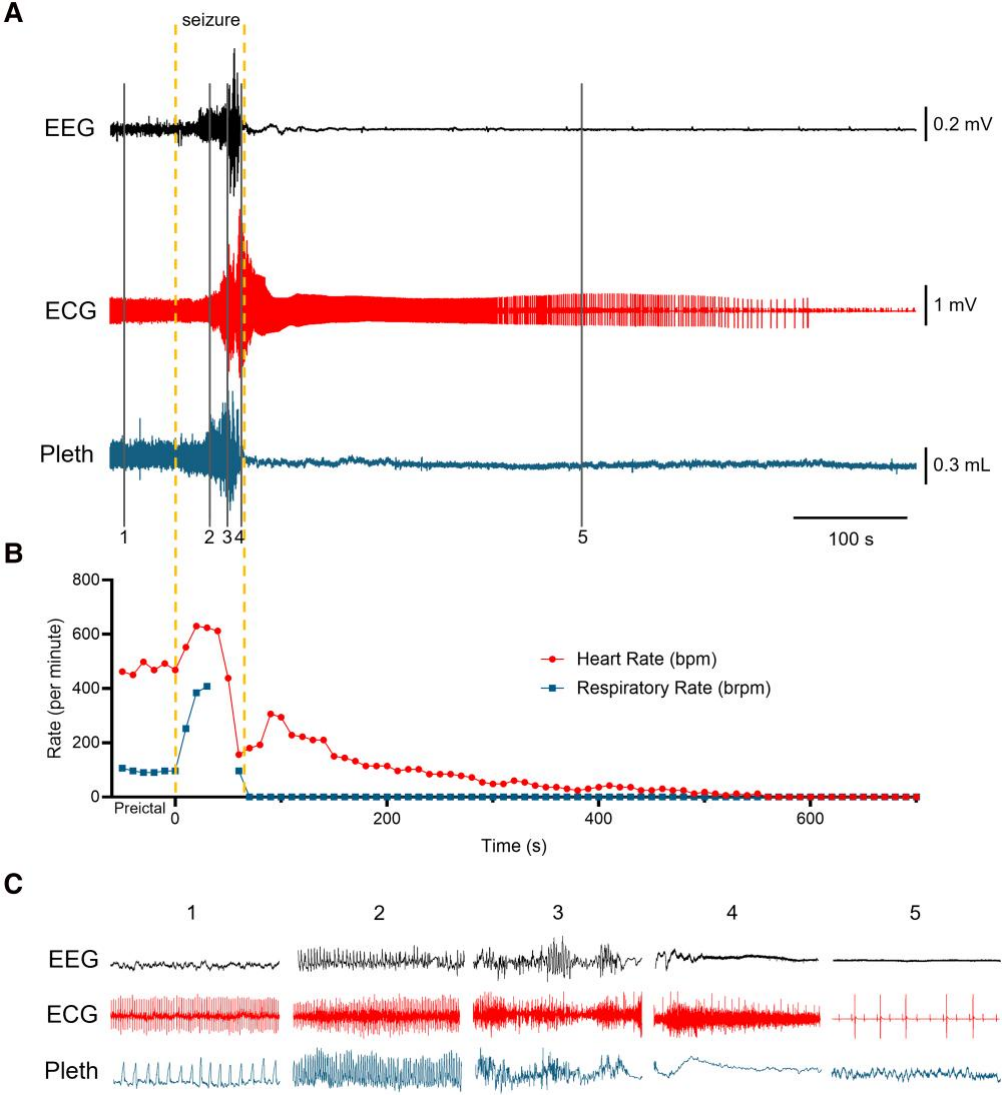
**A SUDEP event in a corticolimbic *Kcna1* conditional knockout mouse.** (**A**) Recording of simultaneous EEG-ECG-pleth activity during a spontaneous seizure in a 30-day old female conditional knockout mouse. *Top,* Trace indicates the neural EEG signal; *middle*, trace represents the cardiac ECG signal; *bottom*, trace shows the respiratory plethysmography (Pleth) signal. Onset and termination of the seizure are indicated by the dotted lines. (**B**) Respiratory (brpm) and heart rates per minute (bpm) corresponding to the recording in **A**. Each dot in the plot represents a 10-s mean value. Time 0 represents seizure onset, and the pre-ictal segment shows the 60-s immediately preceding seizure onset. Some movement artefacts were present in the pleth and ECG signals; therefore, scoring of breaths and heartbeats was done manually as needed. The respiratory rate data points at 40-s and 50-s were omitted, resulting in a discontinuous line, because convulsive seizure activity hindered accurate rate calculations. (**C**) Expanded 10-s traces of EEG, ECG and pleth at the times indicated by the solid lines numbered 1–5 in **A**. The numbers mark the following events: 1, pre-ictal phase; 2, tachypnea with slightly increased heart rate shortly after seizure onset; 3, ataxic-like breathing patterns and mild bradycardia shortly before seizure termination; 4, onset of post-ictal generalized EEG suppression with terminal apnoea and severe bradycardia; 5, extreme post-ictal bradycardia. The high frequency activity in the ECG during portions of the seizure reflects skeletal muscle activity.

When the SUDEP event occurred, the mouse had a generalized seizure lasting slightly longer than average, about 51 s (denoted by the darkened circle in Fig. 2C). This seizure triggered abnormal breathing and cardiac patterns, followed by PGES, ultimately resulting in death (Fig. 5A). Behaviourally, the seizure started with a Straub tail, circling, and forelimb myoclonus, and then escalated to running and bouncing before culminating in tonic hindlimb extension and death (Supplementary Video 1).

Pre-ictally, the mouse had normal cardiorespiratory activity (Fig. 5B and C, inset #1). Shortly after seizure onset, tachypnea became apparent with a mild increase in HR (Fig. 5B and C, inset #2). As the seizure intensified with running and bouncing, respiration became irregular exhibiting ataxic-like breathing patterns. About 5 s after ataxic-like breathing patterns began, the HR slowed, becoming bradycardic (Fig. 5B and C, inset #3). As the seizure ended with tonic hindlimb extension, breathing abruptly stopped and post-ictal apnoea ensued, coinciding with PGES (Fig. 5B and C, inset #4). After PGES onset, both breathing and brain activity did not resume. Moreover, the bradycardia observed during the seizure worsened post-ictally, with the RR intervals getting progressively longer and interspersed with non-conducted P waves indicative of cardiac conduction blocks (Fig. 5B and C, inset #5). Finally, about 8.2 min after the seizure ended, all cardiac activity ceased. These findings suggest that SUDEP in this case was triggered by a generalized tonic-clonic seizure that evoked primary respiratory failure leading to subsequent cardiac arrest.

### Interictal cardiorespiratory function appears normal in corticolimbic conditional knockout mice

**Figure 6:**
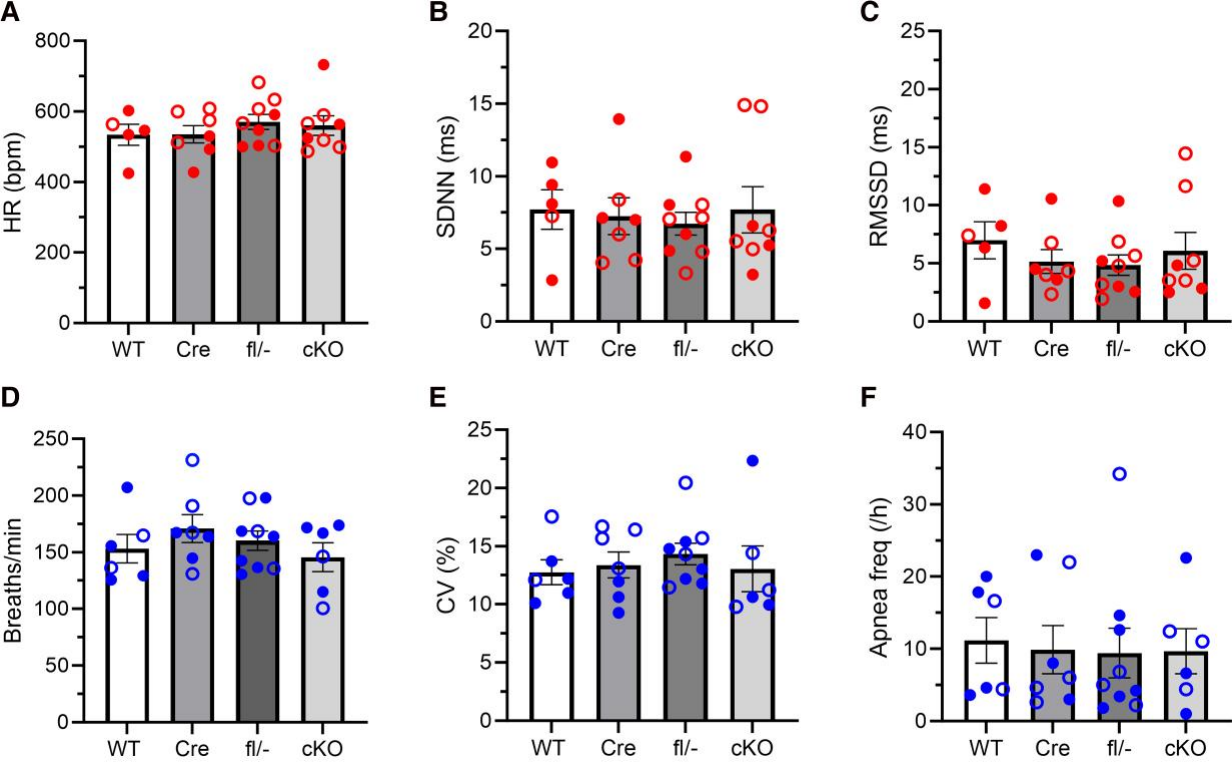
**Corticolimbic conditional knockout (cKO) mice do not display obvious interictal cardiorespiratory dysfunction.** Quantification of interictal cardiac measures for HR (1-way ANOVA, *P* = 0.68) (**A**), SDNN as an estimate of total autonomic activity (1-way ANOVA, *P* = 0.93) (**B**), and RMSSD as an estimate of parasympathetic activity (1-way ANOVA, *P* = 0.26) (**C**) for WT (*n* = 5), Cre (*n* = 7), fl/− (*n* = 9) and cKO (*n* = 7) mice. Quantification of interictal respiratory measures for respiratory rate (1-way ANOVA, *P* = 0.48) (**D**), CV as an estimate of respiratory variability (1-way ANOVA, *P* = 0.81) (**E**), and total apnoea frequency (1-way ANOVA, *P* = 0.98) (**F**) for WT (*n* = 6), Cre (*n* = 7), fl/− (*n* = 9) and cKO (*n* = 5) mice. Total apnoea frequency included post-sigh and spontaneous apnoeas. CV = coefficient of variance. For the data points, closed circles indicate male sex, and open circles indicate female sex. HR = heart rate; SDNN = standard deviation of beat-to-beat intervals; RMSSD = root mean square of successive beat-to-beat differences.

Neural circuits targeted by *Emx1*-Cre play important roles in the regulation of heart and lung function, so we examined ECG and plethysmography recordings for evidence of cardiorespiratory dysfunction during interictal periods that could be indicative of increased SUDEP risk. In ECG recordings, we found no obvious differences in interictal cardiac parameters. Both HR and HR variability appeared similar between conditional knockout mice and controls (Fig. 6A–C). In plethysmography recordings, conditional knockout mice also showed no significant differences in interictal respiration. The conditional knockout mice appeared indistinguishable from control mice in their breathing rate, respiratory variability, and apnoea frequency (Fig. 6D–F). Thus, cardiorespiratory measurements did not reveal any obvious interictal abnormalities that could indicate heightened risk of SUDEP.

## Discussion

This study is the first to identify excitatory neurons of the neocortex and limbic system as critical neural substrates driving seizures and subsequent SUDEP in the *Kcna1* KO mouse model. In addition, our findings suggest that the absence of Kv1.1 in forebrain corticolimbic circuits promotes deleterious ictal cardiorespiratory dysfunction. Notably, we recorded a SUDEP event in a corticolimbic conditional knockout mouse, offering valuable insights into the terminal sequence of events leading to death. During the SUDEP episode, the mouse exhibited a pattern of seizure-induced cardiorespiratory failure resembling clinical cases reported in humans. Our documentation of a SUDEP event, complete with cardiorespiratory data, is significant because such recordings have only been achieved in four other rodent models with spontaneous seizures, and never before in a K^+^ channel model of SUDEP. Surprisingly, we did not observe any obvious interictal cardiorespiratory deficits that could signal susceptibility to SUDEP. Thus, this work narrows the brain circuitry underlying SUDEP in the *Kcna1* KO mouse model to include excitatory corticolimbic neurons while also providing support for seizure-related respiratory impairment as a key mechanism in the pathophysiology of SUDEP.

Our recorded SUDEP event offers a rare glimpse into the terminal sequence of events in a preclinical model. Additionally, it represents the first report of a SUDEP event in a K^+^ channel model with comprehensive cardiorespiratory monitoring. Capturing SUDEP events with corresponding cardiorespiratory data in mouse models with spontaneous seizures is challenging due to the low incidence and unpredictable nature of SUDEP, along with technical challenges in simultaneous biosignal collection.^7^ To our knowledge, recorded SUDEP events with peri-ictal brain, heart and breathing data have only been reported for four other genetic mouse models of chronic epilepsy: *Depdc5* conditional knockout mice deleting the gene in forebrain regions under the *Rbp4*-Cre driver; *Scn1a*^R1407X*/+*^ mice; *Scn8a^R1872W/+^* mice; and *Scn8a^R1872W/+^* mice with the mutation in *Emx1*-Cre targeted forebrain excitatory neurons.^17-19^ These models exhibit a common SUDEP pattern of a generalized tonic-clonic seizure followed by tonic hindlimb extension and PGES. Furthermore, during fatal seizures each model displays ictal apnoea followed by cardiac slowing. Post-ictally, breathing never resumes and HR gradually decreases before terminal cardiac arrest. This pattern of seizure-related respiratory arrest followed by cardiac failure and death has also been observed in mouse models with seizures induced by audiogenic stimulation or maximal electroshock.^20,21^

The SUDEP event we describe here follows a similar pattern, with a generalized tonic-clonic seizure ending in tonic hindlimb extension and exhibiting ictal cardiorespiratory irregularities, where respiratory arrest precedes cardiac failure. However, unlike previous reports in other mouse models, terminal apnoea in our model began post-ictally when EEG seizure activity ceased and PGES was evident. Compared with the *Depdc5* and Na^+^ channel models, our pattern more closely aligns with observations from the human MORTEMUS study. In that study, cardiorespiratory collapse consistently occurred post-ictally, with terminal apnoea preceding asystole.^6^ One possible explanation for this close resemblance is that *Kcna1* deletion models temporal lobe epilepsy, the predominant type of epilepsy represented in patients in the MORTEMUS study.^6^ In contrast, the other four SUDEP mouse models exhibit seizures characteristic of other types of epilepsy, such as developmental epileptic encephalopathy and familial focal epilepsy.^18,19,22-24^ Collectively, mouse models and human cases show a common theme of breathing dysfunction usually preceding cardiac abnormalities, suggesting respiratory factors may drive fatal cardiorespiratory collapse. However, this remains to be conclusively demonstrated. An alternative explanation could be that seizures independently initiate respiratory and cardiac dysfunction, with differing time courses favouring the appearance of apnoea first. Determining the relative importance of respiratory and cardiac mechanisms in mediating death will be imperative for developing proper risk diagnostic and preventative strategies.

The presence of seizure-induced cardiorespiratory dysfunction in corticolimbic conditional knockout mice highlights the potential importance of forebrain corticolimbic structures in the regulation of cardiac and respiratory function. Although the brainstem is generally thought of as the primary locus of cardiorespiratory control, corticolimbic regions such as the cortex, hippocampus and amygdala also exert significant influence on heart and lung function through direct communication with brainstem nuclei. In people with epilepsy, the spread of seizures to or direct electrical stimulation of the amygdala and/or hippocampus can induce apnoea, often without air hunger perception, potentially increasing susceptibility to SUDEP.^25-29^ From a cardiac standpoint, epileptic discharges in or electrical stimulation of the amygdala and hippocampus can produce tachy- or bradycardia, indicating forebrain influence over cardiac autonomic control.^30-32^ Thus, seizures can hijack the normal autonomic regulatory roles of corticolimbic circuits resulting in cardiorespiratory dysfunction and potentially increasing the risk of SUDEP.

An important limitation of our study is that while *Emx1*-Cre is well known for its selectively targeting corticolimbic neurons, recent reports indicate its expression in some vagal afferents as well.^17^ Therefore, it is possible that Kv1.1 deficiency in vagal afferents could contribute to the seizure-related cardiorespiratory dysfunction in our model. However, neuron-specific *Kcna1* conditional knockout mice, which lack *Kcna1* deletion in vagal afferents, display spontaneous seizures with ictal cardiorespiratory dysfunction similar to corticolimbic conditional knockouts.^10^ This implies that these phenotypes originate from the brain. Furthermore, interictal cardiorespiratory abnormalities were absent in corticolimbic conditional knockout mice, suggesting that any potential influence of vagal afferent hyperexcitability is not sufficient to impair basal cardiorespiratory function. Finally, our breeding strategy resulted in conditional knockout mice that possess both a global *Kcna1* null allele and a conditionally deleted allele. The presence of the global null allele theoretically reduced Kv1.1 levels by about half in tissues not targeted by *Emx1*-Cre, which may have also influenced the observed phenotypes.

Our discovery that the absence of Kv1.1 in corticolimbic circuits underlies epilepsy brings together previous findings that hinted at the crucial role of these structures in facilitating seizures resulting from Kv1.1 deficiency. Protein studies have shown that Kv1.1 is abundant throughout corticolimbic brain regions, including the hippocampus (CA3, hilus and dentate gyrus),^11,33^ amygdala (including the basolateral amygdala),^34^ and neocortical pyramidal cells.^11,35,36^ In the absence of Kv1.1 in these structures, brain slice recordings have revealed neuronal hyperexcitability and epileptiform-like activity, indicating a low threshold for epileptic activity.^8,37-40^ Moreover, spontaneous seizures in *Kcna1* KO mice exhibit behaviours characteristic of limbic seizures induced by kainate or kindling, such as facial twitching, head nodding, rearing and falling, and forelimb and hindlimb clonus.^8,33,41^ EEG recordings using depth electrodes show that seizures in *Kcna1* KO mice sometimes originate in the hippocampus before spreading to the cortex.^33^ Furthermore, spontaneous seizures in *Kcna1* KO mice significantly activate Fos, a protein marker of intense neuronal activity, in the dentate hilus and basolateral amygdala, indicating ictal recruitment of these brain regions.^22^ Additionally, the hippocampus and amygdala of *Kcna1* KO mice exhibit extensive gliosis, suggesting potential brain injury due to repeated seizures.^33,34^ Magnetic resonance imaging of *Kcna1* KO brains reveals significant enlargement of the hippocampus and ventral cortex, including the amygdala, suggesting brain volume increases associated with seizures.^42^ Thus, our study demonstrates for the first time that Kv1.1 deficiency in corticolimbic circuits is a primary driver of seizures in *Kcna1* KO mice, consolidating multiple lines of evidence from previous studies.

Corticolimbic conditional knockout mice exhibited premature death, seizures and cardiorespiratory phenotypes that were generally less severe overall than those observed in neuron-specific *Kcna1* conditional knockout and global *Kcna1* KO mice. Corticolimbic conditional knockout mice displayed lower SUDEP rates, with ∼75% of animals surviving to 100 days old compared with only 45% and 23% of neuron-specific conditional knockout and global KO mice, respectively.^10,13^ The onset of SUDEP in corticolimbic and neuron-specific conditional knockout mice occurred at approximately the same age, around 3–4 weeks old, which was about a week later than SUDEP onset in global KOs. Although average seizure frequencies and durations appeared similar across the different *Kcna1* models, long-duration seizures (>60 s) were rare in corticolimbic conditional knockout mice (about 6% of the time) compared to 17% and 35% in neuron-specific conditional knockout and global KO mice, respectively.^10,13^ During seizures, all *Kcna1* models exhibited varying degrees of ictal cardiorespiratory abnormalities, with respiratory dysfunction being more prevalent and consistently preceding cardiac dysfunction.^10,13^ While cardiac conduction blocks/sinus pauses and bradycardia were relatively rare in neuron-specific conditional knockout mice (occurring in <10% of seizures), they were more prevalent in corticolimbic conditional knockout mice, present in about 20–30% of seizures. However, the most significant difference between corticolimbic conditional knockout mice and the other *Kcna1* models was the absence of interictal cardiorespiratory dysfunction. Unlike neuron-specific conditional knockout and global KO mice, which showed increased HR variability and a nearly complete lack of apnoeas between seizures,^10,13^ corticolimbic conditional knockout mice exhibited no obvious cardiorespiratory abnormalities during interictal periods. This suggests that Kv1.1 may not be necessary in corticolimbic circuits or vagal afferents for proper baseline cardiorespiratory function. Thus, corticolimbic conditional knockout mice displayed seizure characteristics generally similar to neuron-specific conditional knockout and global KO mice, but with lower mortality rates, and the most marked difference lies in their relative lack of interictal cardiorespiratory dysfunction.

In summary, our findings implicate excitatory forebrain neurons, with a possible influence of vagal afferents, as primary drivers of epilepsy and subsequent SUDEP in the *Kcna1* mouse model. The similarity of our recorded SUDEP event to observations in humans underscores the significance of corticolimbic conditional knockout mice as valuable tools for understanding the pathophysiological mechanisms underlying SUDEP. A critical objective of future research will be to capture cardiorespiratory data surrounding additional SUDEP events across various models to ascertain whether the observed terminal pattern of seizure-induced fatal apnoea preceding cardiac failure is a consistent feature of SUDEP cases or if multiple mechanisms can lead to the same outcome. Furthermore, it will be essential to investigate the potential contributions of other neuronal subpopulations, such as those in the brainstem, to seizures, cardiorespiratory dysfunction, and SUDEP in the *Kcna1* model and other preclinical models.

## Supplementary Material

fcae444_Supplementary_Data

## Data Availability

The data that support the findings of this study are available from the corresponding author upon reasonable request.
