# Supplementary material for: Seizures and premature death in mice with targeted Kv1.1 deficiency in corticolimbic circuits
Source: Brain Commun. 2025 Jan 16;7(1):fcae444. doi: 10.1093/braincomms/fcae444 (PMC11735082; doi:10.1093/braincomms/fcae444)
Supplement: fcae444_Supplementary_Data [file fcae444_supplementary_data.zip › Supplementary Materials.pdf]

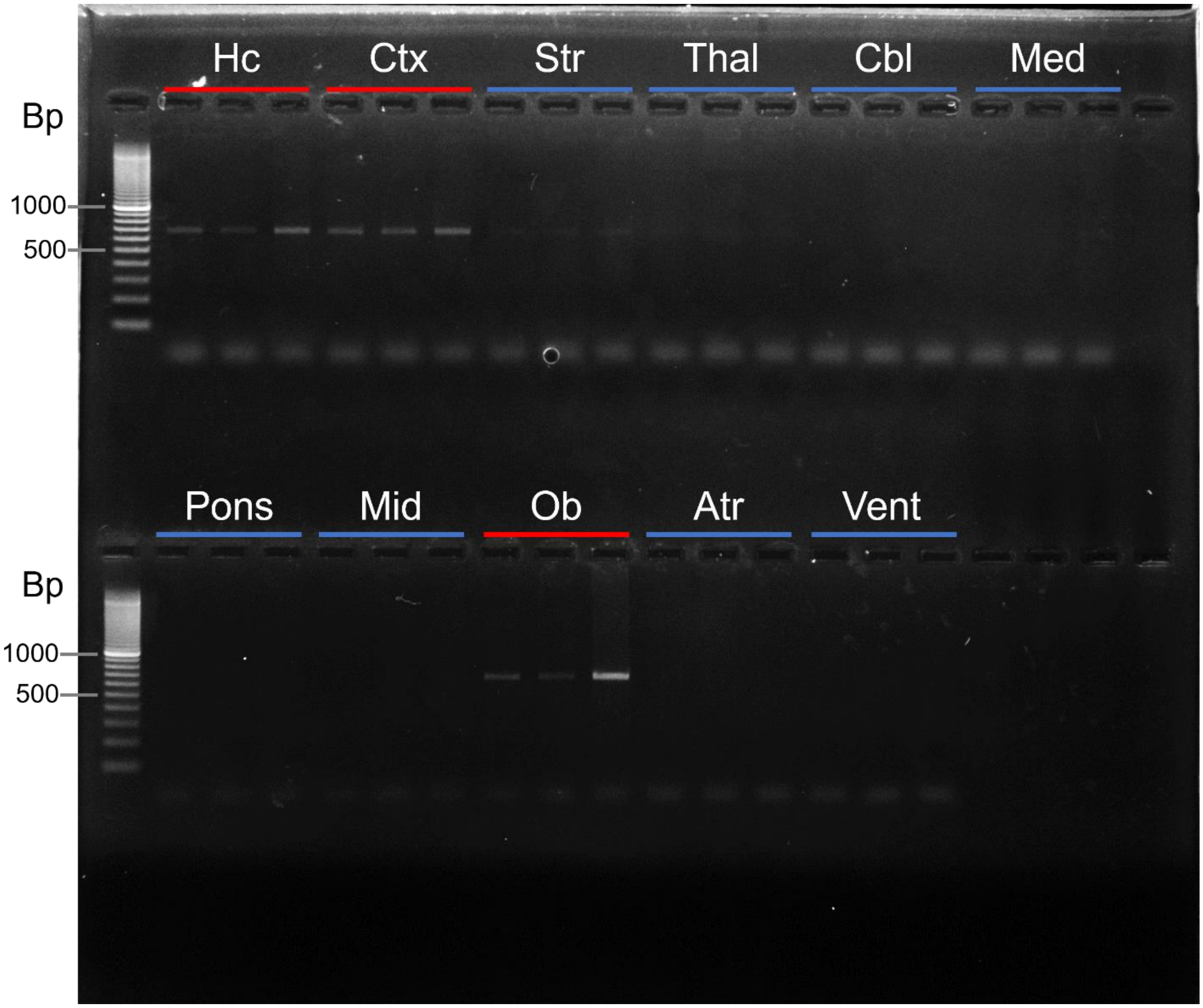

Supplementary Figure 1. Uncropped version of PCR gel image from Figure 1A in the main text.

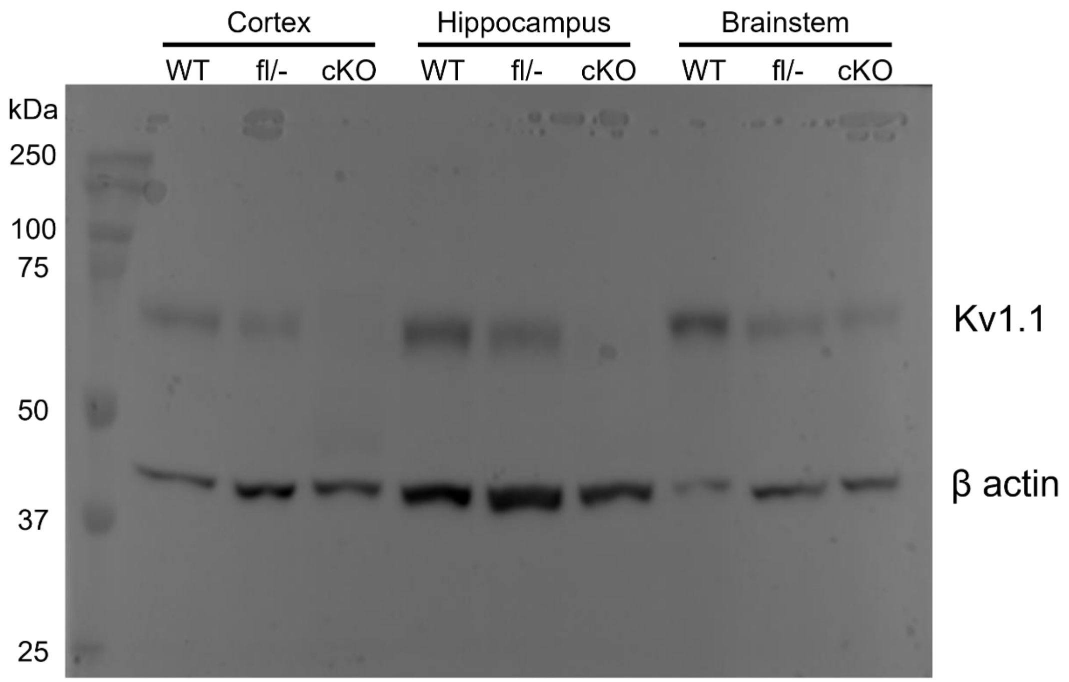

Supplementary Figure 2. Uncropped version of Western blot image from Figure 1B in the main text.
