# Supplementary material for: Seizures and premature death in mice with targeted Kv1.1 deficiency in corticolimbic circuits
Source: Brain Commun. 2025 Jan 16;7(1):fcae444. doi: 10.1093/braincomms/fcae444 (PMC11735082; doi:10.1093/braincomms/fcae444)
Supplement: fcae444_Supplementary_Data [file fcae444_supplementary_data.zip › Supplementary Video 1 Legend.docx]

**Supplementary Video 1.** Sudden unexpected death in epilepsy in a 30-d old female corticolimbic conditional knockout mouse. The top blue trace shows the respiratory pleth signal, the middle black traces indicate the left and right neural EEG signals, and the bottom red signal represents the cardiac ECG signal. Abbreviations: Pleth, plethysmography; EEG, electroencephalography; ECG, electrocardiography.
